# Novel Visualization of Building Earthquake Response Recorded by a Dense Network of Sensors

**DOI:** 10.3390/s25020417

**Published:** 2025-01-12

**Authors:** Lichiel Cruz, Maria I. Todorovska, Mihailo D. Trifunac, Alimu Aihemaiti, Guoliang Lin, Jianwen Cui

**Affiliations:** 1 Department of Civil Engineering, Tianjin University, Tianjin 300350, China; lichiel.cruz@gmail.com (L.C.); alimahmat@tju.edu.cn (A.A.); 2 Department of Civil and Environmental Engineering, University of Southern California, Los Angeles, CA 90089-2531, USA; trifunac@usc.edu; 3 Yunnan Earthquake Agency, Kunming 650224, China; linguoliang1986@126.com (G.L.); hxmcjw@163.com (J.C.)

**Keywords:** dynamic surface deformation, biharmonic spline interpolation, visualization of a dense network seismic data, animation of recorded building earthquake response, Tongde Plaza Yue Center (TPYC) testbed structure

## Abstract

The strong motion records collected in full-scale structures provide the ultimate evidence of how real structures, in situ, respond to earthquakes. This paper presents a novel method for visualization, in three dimensions (3D), of the collective motion *recorded* by a dense array of sensors in a building. The method is based on one- and two-dimensional biharmonic spline interpolation of the motion recorded by multiple sensors on the same or multiple floors. It is demonstrated on novel data that have been recorded recently in a 50-story skyscraper, uniquely instrumented with multiple triaxial accelerometers per floor, approximately at every five floors above ground and at two basement levels, and with rotational seismometers and two borehole arrays measuring the motion of the soil very near the building foundation. The method is computationally efficient and suitable for real-time application and rapid assessment of structural health. The animations provide invaluable insight into the 3D structural response of the building as a whole, including wave propagation through the structure and the interplay between translations and rotations, which will be useful for testing existing and developing new methods for structural health monitoring of buildings and for the further development of building design codes. Animations of selected earthquakes can be found on YouTube at @TPYC-seismic.

## 1. Introduction

**Figure 1:**
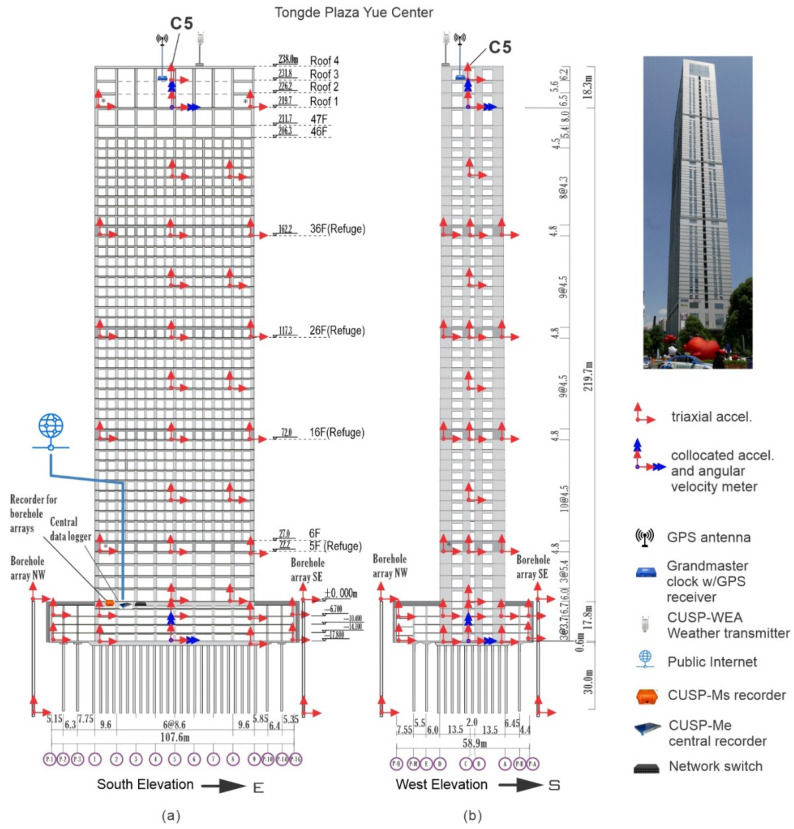
Tongde Plaza Yue Center (TPYC) vertical elevations showing the location of the sensors. (**a**) East elevation and (**b**) West elevation.

We present a novel method for visualizing, in 3D space and in time, the earthquake response of a building, as a whole, based on the *recorded* response by a dense array of sensors. We demonstrate the method on an instrumented 50-story skyscraper (Figure 1) [1] and present an animation of the actual response recorded in the building during a recent earthquake, providing visual evidence of how an actual building, in situ, responds to an earthquake. The method involves representation of the floor slab motion as a dynamic surface deformation defined by two-dimensional (2D) biharmonic splines [2,3,4,5,6,7,8]. The method can be extended to other types of densely instrumented structures, like bridges and dams.

Earthquake records from instrumented structures have been essential for the evolution of earthquake engineering; a review of the progress in sensor technologies and deployment since the 1930s can be found in [9]. The first comprehensive set of earthquake records in structures was generated by the 1971 San Fernando earthquake in California and presented visually in terms of plots of time histories of recorded acceleration, velocity, and displacement of the individual records and the corresponding Fourier and response spectra [10]. Such a form of visual presentation has become the standard and is currently disseminated over the internet [11,12,13,14]. To maximize the coverage of different types of construction in different seismic zones, the strong motion arrays in buildings have been sparse, with only a few instrumented floors and with uniaxial or biaxial accelerometers recording only horizontal responses. Methods have been proposed to reconstruct the motion at the non-instrumented floors directly by interpolation [6,7,8], a model of the structure [15,16], or a combination of the two [17,18]. The number of densely instrumented buildings has been slowly but steadily growing [19,20,21,22,23,24,25,26,27,28,29]. Yet, even in these buildings, only the horizontal components of the floor responses are recorded, while vertical motions are typically recorded only in the basement.

Rare examples of densely instrumented buildings with sensors recording vertical floor motion are the Los Angeles 52-story building, instrumented with one triaxial accelerometer at practically every floor [25], and the case study in this paper, a 50-story building with instrumented approximately every fifth floor above ground, but by multiple triaxial accelerometers, and two basement levels instrumented each with seven triaxial accelerometers, which enables reconstruction of the motion of the floor slabs [1]. Analyses of vertical vibrations recorded in these two buildings can be found in [30,31].

The case study in this paper is also a rare example of a full-scale building permanently instrumented with rotational seismometers. One of them is in the basement and the other one is on the 48th floor (Figure 1), the latter being used to reconstruct the 48th floor slab motion, as shown in the next section. Recent articles on rotational sensors and their applications to recording ground motion and structural response can be found in [28,29,30,31,32,33]. An analysis of earthquake records of rotations recorded in the 50-story case study can be found in [34]. Finally, unique elements of the sensor array in the case study are two borehole arrays installed close to the building, with one sensor at the ground surface and another one at the depth reached by the piles (~50 m), the motions of which are also animated.

In this paper, biharmonic spline interpolation is used to reconstruct the floor slab motion, separately for each component of motion, at a sequence of time instances, creating an animation. To the knowledge of the authors, this is the first case of visualization of floor deformations of a building during an earthquake, directly from observed data, not involving a model of the structure, and the first visualization of a 3D response of a building directly from *recorded* response.

Biharmonic spline interpolation is particularly effective in reconstructing smooth, continuous surfaces from sparse and irregularly spaced data points. It has been previously applied, e.g., to interpolation of satellite altimeter data [2], surface reconstruction in computer graphics [3], mapping solar radiation [5], and reconstruction of horizontal floor motion in sparsely instrumented buildings [6,7,8].

This paper is organized as follows. Section 2 presents a brief summary of the case study, the data, and the methodology. The theory of biharmonic spline interpolation for observations distributed spatially in 1D and 2D is reviewed and the steps of creating the animation for the case study are deliberated. Section 3 presents the results in the form of selected snapshots of the animated response recorded during an earthquake and a pointer to a YouTube channel where similar animations for other earthquakes have been posted. Finally, Section 4 presents a discussion and the conclusions of this study.

## 2. Materials and Methods

### 2.1. Building and Data

**Figure 2:**
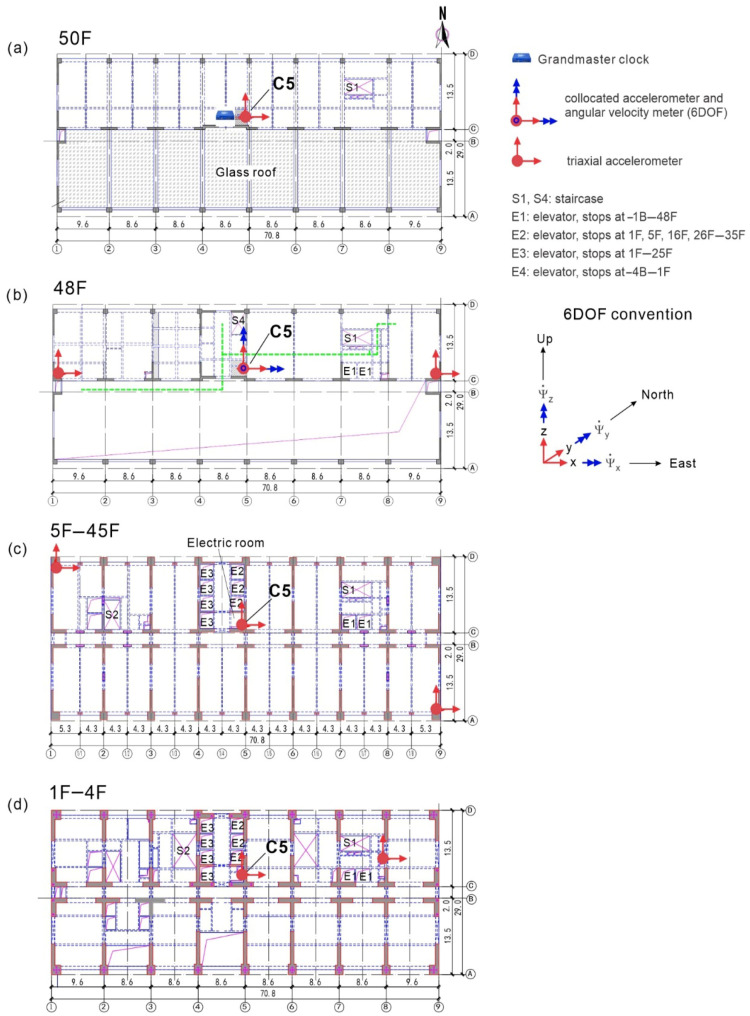

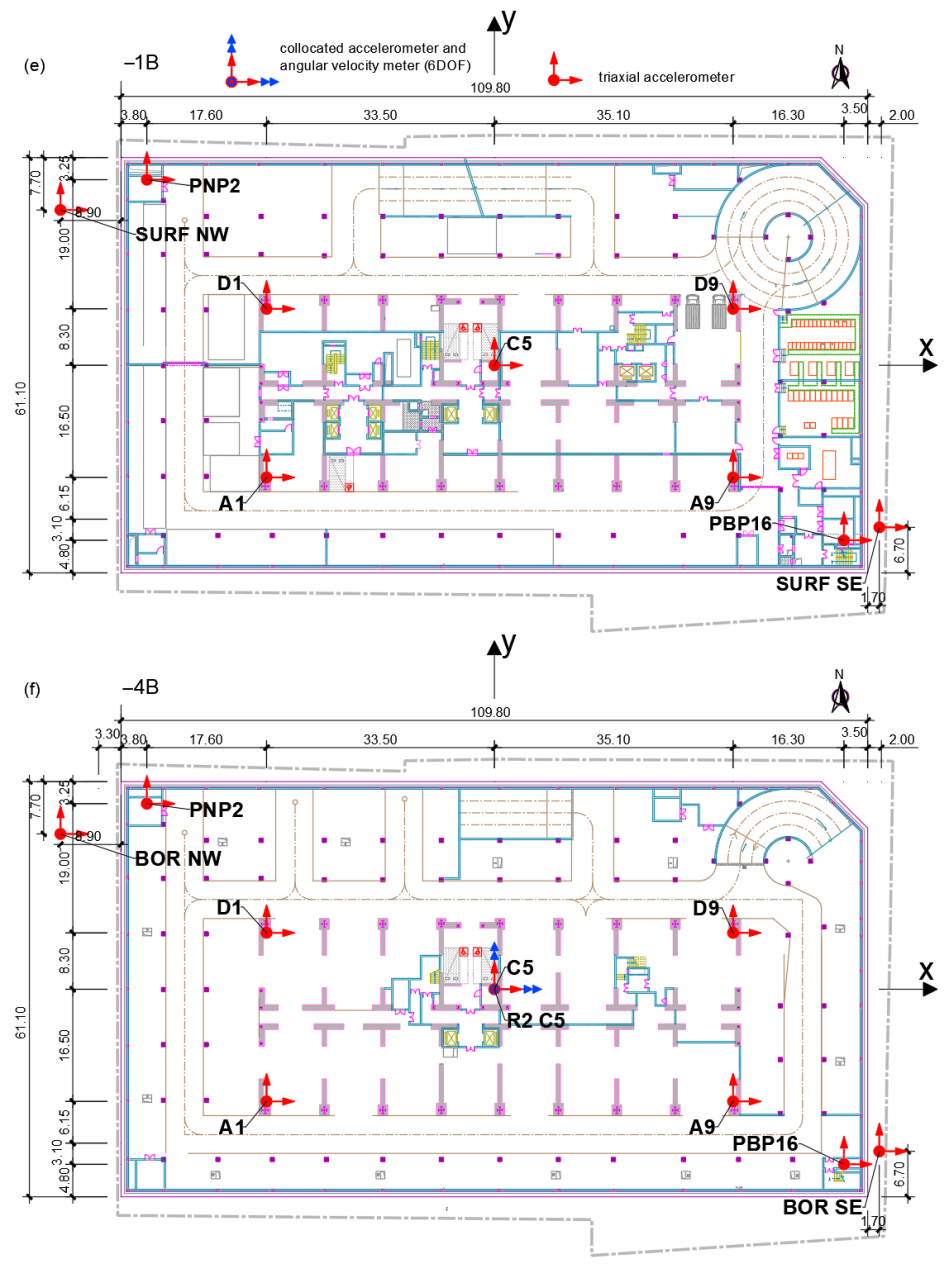
Structural floor layout of TPYC: (**a**) floor 50F, (**b**) floor 48F, (**c**) floors 5F to 45F, (**d**) floors 1F to 4F, (**e**) floor −1B and (**f**) floor −4B.

**Figure 3:**
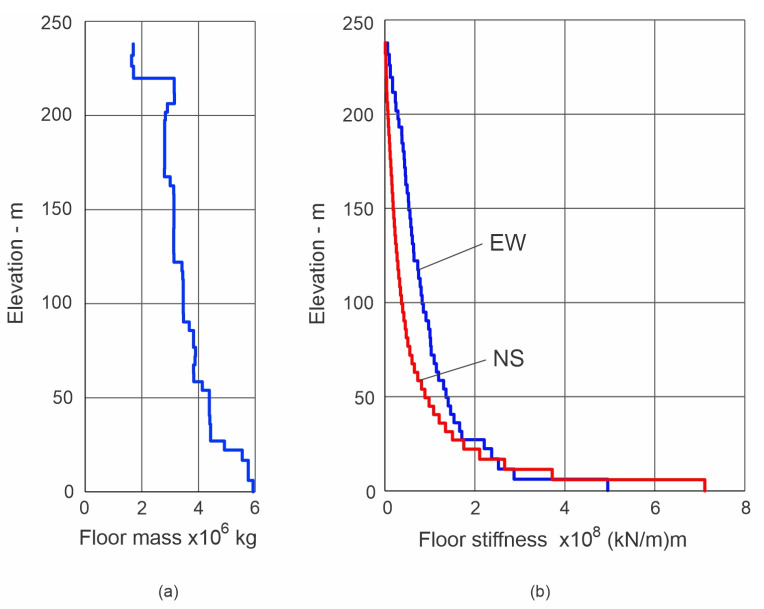
Distribution of floor masses (part (**a**)) and lateral stiffnesses (part (**b**)) of the TPYC building along the height in the north–south (NS) and east–west (EW) directions, obtained from a detailed finite element model of the tower, fixed at the ground floor [30].

The building is known as Tongde Plaza Yue Center (TPYC) and is located in Kunming, Yunnan Province of China. It is a reinforced concrete (RC) construction, with 50 floors (238 m) above and 4 floors (17.8 m) below ground (Figure 1 and Figure 2), comprising a tower and a basement proper encompassing the tower below ground. The tower is 70.8 × 29 m^2^ in plan, approximately symmetrical in both horizontal directions, while the basement is 105.6 × 58.9 m^2^ in plan and 17 m deep. They are both supported by a raft foundation and 30 m long RC friction piles. The building use is commercial, and the basement is used for parking. Figure 1 shows the east and south elevations and Figure 2 shows typical floor layouts, of the floors above ground (parts (a) to (d)) and of basements −1B and −4B (parts (e) and (f)). The tower is stiff near the base and flexible near the top, as illustrated in Figure 3, which shows the distribution, along the height, of the floor masses and floor lateral stiffnesses in the north–south (NS) and east–west (EW) directions. These distributions were obtained from a detailed finite element model of the tower, fixed on the ground floor [30], and are presented in this paper to facilitate the interpretation of the animated building response, presented in the Results section, which is entirely based on recorded response.

The basic dynamic and wave properties of the structure, as determined from ambient vibration data [30], are as follows. The fundamental frequencies are $f_{1}^{NS}=$ 0.27 Hz (1st NS mode), $f_{1}^{T}=$ 0.37 Hz (1st torsional mode), $f_{1}^{EW}=$ 0.39 Hz (1st EW mode), and $f_{1}^{Up}=$ 3.23 Hz (1st vertical mode). The wave propagation in the structure was found to be dispersive, with vertical phase velocities in the band 0–3.2 Hz of about $c_{z}^{EW}=$ 417 m/s and $c_{z}^{NS}=$ 450 m/s, associated with deformations in the NS and EW directions, respectively. Further, based on the fundamental vertical frequency ($f_{1}^{Up}=$ 3.23 Hz), the corresponding phase velocity associated with vertical deformations is about $c_{z}=$ 2800 m/s. These quantities are amplitude dependent and have lower values during earthquake response.

The instrumentation includes 26 triaxial accelerometers distributed across eleven levels of the tower, located at floors 1F, 5F, 10F, 16F, 21F, 26F, 31F, 36F, 42F, 48F, and 50F. Additionally, fourteen triaxial accelerometers are installed at two basement levels (−1B and −4B). The two borehole arrays are equipped each with two accelerometers, one at the surface and the other one at 48.4 m depth. The accelerometer array is complemented by two rotational seismometers, located at −4B and 48F, and a weather receiver located on the roof. The sensor layout is shown in Figure 1 and Figure 2.

All accelerometers are Class B Micro-Electro-Mechanical Systems (MEMS) servo silicon sensors (EQR120) [1], with a dynamic range of 128 dB between 0.1 and 20 Hz and 120 dB between 0.1 and 100 Hz. Those inside the tower and basement have a recording range of ±3*g* and threshold of 2.4 × 10^−6^*g*, while the two pairs of the borehole arrays have a recording range of ±5*g* and threshold of 3.9 × 10^−6^*g*. The rotational seismometers are R2 angular velocity meters, with a resolution 6 × 10^−8^ rad/s at 1 Hz [30,31,32,33,34,35,36,37,38]. All accelerometers have built-in 24-bit Σ-Δ type analog-to-digital (A/D) conversion with 31-bit resolution and signal to noise ratio (SNR) of 130 dB at 200 Hz, 133 dB at 100 Hz, and 136 dB at 50 Hz. The sampling frequency was set to 200 Hz, corresponding to a Nyquist frequency of 100 Hz.

Since January 2021, more than 35 earthquakes have been recorded in the building, with epicenters in Yunnan and Sichuan Provinces of China and neighboring Burma, Laos, and India. The earhqukaes had magnitudes $2.1\leq M_{s}\leq 6.6$, epicentral distances 10≤R≤1008 km, and focal depths between 9 and 128 km. The recorded motions were small. The largest motions were recorded during the M6.4 Yangbi, Dali, Yunnan earthquake of 21 May 2021 (R=295 km), and the M6.8 Kanding, Garze, Sichuan earthquake of 5 September 2022 (R=506 km). The peak ground horizontal and vertical accelerations recorded on the ground surface near the south-east corner of the basement were $a_{H}^{\max}=$ 1.84 × 10^−3^*g* and $a_{V}^{\max}=$ 1.02 × 10^−3^*g* (Yangbi), and $a_{H}^{\max}=$ 1.30 × 10^−3^*g* and $a_{V}^{\max}=$ 0.68 × 10^−3^*g* (Kanding) (1*g* = 9.80665 m/s^2^), while the response of the building (at the 48th floor) was within 9 × 10^−3^ *g*, and no damage was observed. More details about the structure, soil, structural health monitoring system, and recorded earthquakes can be found in [1,30,39].

### 2.2. Methodology

**Figure 4:**
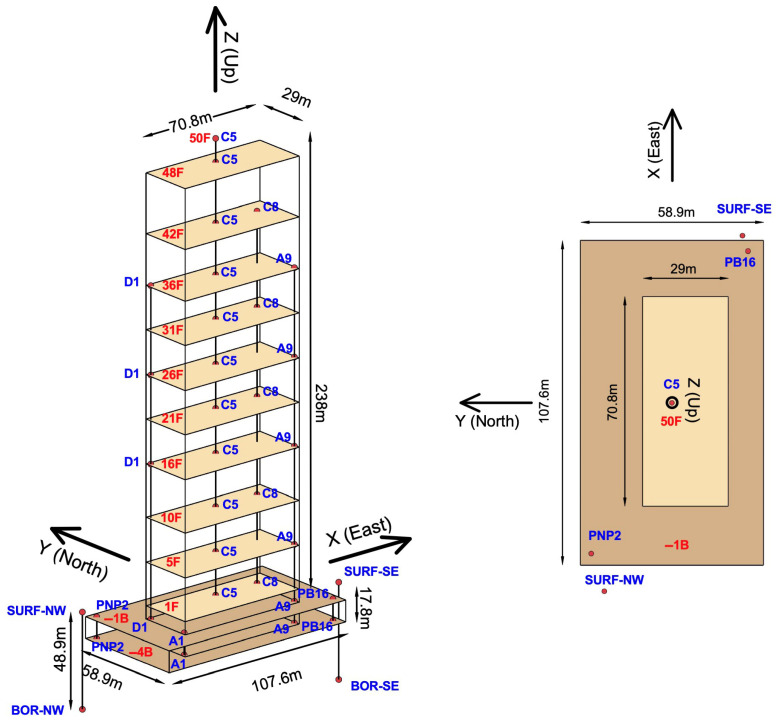
Model (side and top views) of the TPYC used to build the animation of the recorded response, showing the slabs of the instrumented floors, sensor locations, and the global X−Y−Z coordinate system.

The animation of the TPYC motion is built based on the model in Figure 4, which shows the slabs of the instrumented floors and the sensor locations. The vertically aligned sensor locations are connected by straight line segments. The corners of the surfaces representing the slabs are also connected by line segments to outline the boundaries of the structure. The motion of the model is described relative to the X−Y−Z global reference system. Its origin coincides with the location of the sensor on the first floor at column line C5, which is near but not exactly at the center of the floor. The X-, Y- and Z-axes point East, North, and upward.

Table 1 shows the coordinates of the sensors relative to the X−Y−Z coordinate system. The locations are named by the column line near which the sensor is located (Figure 1 and Figure 2). The orientation of the components of the R2 rotational seismometers is shown in Figure 2b.

### 2.3. Dynamic Surface Representation by 2D Biharmonic Splines Using Data from Triaxial Sensors

Historically, the term “spline” has referred to flexible strips used by draftsmen to draw smooth curves between points. Biharmonic splines represent an extension of this concept to higher dimensions and are valuable in applications where data may be sparse or noisy, and a smooth approximation is desired [2,3]. In one and two dimensions, the biharmonic splines are cubic polynomials that have minimum curvature and continuous first and second derivatives. It has been shown that, in all dimensions, the biharmonic splines have minimum curvature satisfying the constraints [40].

**Figure 5:**
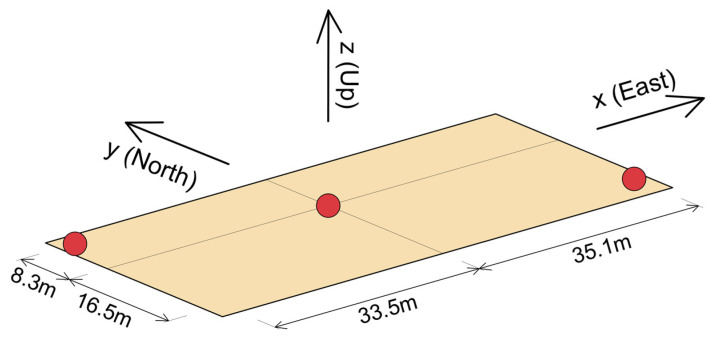
Convention for the directions of the 6DOF motion.

Let us consider an instrumented floor that is at an elevation $Z_{floor}$ from the ground surface, and let x−y−z be a fixed local coordinate system, which has its origin at location C5 on that floor (Figure 5). Then, in the global coordinate system, the points on the slab have coordinates X=x, Y=y and $Z=Z_{floor}$.

Let r=(x,y) be a position vector describing the location of an arbitrary point on the slab in the local coordinate system and let $u(r,t)=\left(u_{x},u_{y},u_{z}\right)(r,t)$ be a three-dimensional vector describing the displacement of that point at time t relative to its position at rest. We wish to express u(r,t) at an arbitrary time instant t as a smooth function of the spatial coordinates such that satisfies the following constraints:

(1) $$u(r_{j},t)=\left(u_{j,x},u_{j,y},u_{j,z}\right)(t),\;j=1,\ldots ,N.$$

Assuming small displacements, we represent each component u(r,t) individually as a biharmonic spline satisfying the corresponding component of the constraints.

Let w(r) be any one of the components of u(r,t) at time t and let $w(r_{i})=w_{i}$ be its constraints. Then, w(r) must satisfy the biharmonic equation and the following constraints:

(2) $$\begin{array}{l} \nabla^{4}w\left(r,t\right)=\sum_{j=1}^{N}\alpha_{j}\delta (r-r_{j});\;\nabla^{4}\equiv {\left(\frac{\partial^{2}}{\partial x^{2}}+\frac{\partial^{2}}{\partial y^{2}}\right)}^{2}\\ \;w(r_{j})=w_{j},\;j=1,\ldots ,N \end{array}$$

in which $\nabla^{4}$ is the biharmonic operator and $\delta (r-r_{j})$ is the Dirac delta function [2]. The solution can be represented as a linear combination of the Green’s functions $\phi \left(r-r_{j}\right)$ of the biharmonic operator, as shown below:

(3) $$w\left(r,t\right)=\sum_{j=1}^{n}\alpha_{j}\phi (r-r_{j}).$$

The Green’s functions are the impulse response functions of the operator and satisfy the following expression:

(4) $$\nabla^{4}\phi (r-r_{j})=\delta (r-r_{j}).$$

In two dimensions [2],

(5) $$\phi (r-r_{j})={\left|r-r_{j}\right|}^{2}\left(\ln\left|r-r_{j}\right|-1\right).$$

Both ϕ(r) and its gradient are continuous everywhere, including at r=0. The unknown coefficients $\alpha_{j}$ can be obtained by substituting into the constraints the representation of w(r) from Equation (3), which leads to a linear system of equations:

(6) $$\sum_{j=1}^{N}\alpha_{j}\;\phi (r_{i}-r_{j})=w_{i},\;i=1,\ldots ,N.$$

The system of equations in (6) can be written in matrix form as follows:

(7) $$\Phi_{N\times N}\alpha_{N\times 1}=w_{N\times 1}$$

in which Φ has entries $\Phi_{ij}=\phi (r_{i}-r_{j})$, $\alpha ={\left(\alpha_{1},\ldots ,\alpha_{N}\right)}^{T}$ and $w={\left(w_{1},\ldots ,w_{N}\right)}^{T}$, and is solved for α. The system has a stable solution if no two rows or columns of Φ are the same, which requires that the points are well distributed on the slab. In the case of many available measurement points that are “noisy”, it is desirable to reduce the order of the system using singular value decomposition.

Green’s function matrix Φ is the same for all three components of motion and does not depend on time. Therefore, it is precomputed and reused. After the coefficients for all three degrees of freedom, $\alpha_{x}$, $\alpha_{y}$ and $\alpha_{z}$, have been computed at a time instance t, the displacement components are computed at that instance on a dense grid of uniformly spaced points on the slab, $r_{k}=(x_{k},y_{k})$

(8) $$\begin{array}{l} u_{x}(r_{k};t)=\sum_{j=1}^{N}\alpha_{x,j}(t)\;\phi (r_{k}-r_{j}),\;k=1,\ldots ,K\\ u_{y}(r_{k};t)=\sum_{j=1}^{N}\alpha_{y,j}(t)\;\phi (r_{k}-r_{j}),\;k=1,\ldots ,K\\ u_{z}(r_{k};t)=\sum_{j=1}^{N}\alpha_{z,j}(t)\;\phi (r_{k}-r_{j}),\;k=1,\ldots ,K \end{array}.$$

Then, the position of the points on the grid at a time t in the absolute coordinate system is computed as follows:

(9) $$R_{k}(t)=(x_{k}+u_{x}(x_{k},y_{k};t),y_{k}+u_{y}(x_{k},y_{k};t),Z_{floor}+u_{z}(x_{k},y_{k};t)),\;k=1,\ldots ,K$$

plots of which show how the slab deforms in time during the earthquake shaking.

### 2.4. Dynamic Surface Representation by a Plane Using Data from a 6DOF Sensor

At the 48th floor, the motion was recorded by a 6DOF sensor (collocated accelerometer and R2 rotational seismometer), located at the origin of the local coordinate system. Let $r={\left(x,y,0\right)}^{T}$ be the position vector of the points on the slab and $u_{0}(t)=(u_{x},u_{y},u_{z})(t)$ and $\Psi_{0}(t)=(\psi_{x},\psi_{y},\psi_{z})(t)$ be the displacements and angles of rotation recorded at the origin, r=0. Then, a plane can be fitted in the recorded 6DOF motions.

The displaced position of the points on the plane can be computed by adding to the translation at r=0, $u_{0}(t)$ displacements resulting from a sequence of rotations about the x-, y-, and z-axes. The latter are linear transformations represented by the following rotation matrices:

(10) $$R_{x}=\left[\begin{array}{ccc} 1 & 0 & 0\\ 0 & \cos\psi_{x} & -\sin\psi_{x}\\ 0 & \sin\psi_{x} & \cos\psi_{x} \end{array}\right]$$

(11) $$R_{y}=\left[\begin{array}{ccc} \cos\psi_{y} & 0 & \sin\psi_{y}\\ 0 & 1 & 0\\ -\sin\psi_{y} & 0 & \cos\psi_{y} \end{array}\right]$$

(12) $$R_{z}=[\begin{array}{ccc} \cos\psi_{z} & -\sin\psi_{z} & 0\\ \sin\psi_{z} & \cos\psi_{z} & 0\\ 0 & 0 & 1 \end{array}].$$

Then, the positions of the points on the slab at a time t in the local coordinate system are as follows:

(13) $$u(r;t)=R_{x}(t)R_{y}(t)R_{x}(t)r+u_{0}(t).$$

We can use the fitted plane to represent the slab motion at the 48F but also to construct constraints for a biharmonic spline representation of that slab, as shown in the next section, as for the other floors.

### 2.5. Constructing Additional Constraints for the Slab Motions

The accuracy and stability of biharmonic spline interpolation depend on the number of constraints (measurement points) and their spatial distribution. This number for the slab motion in our case study is limited to two or three in the tower above ground and to 7 in the basement, and, on the 48th floor, there is only one 6DOF measurement. However, additional constraints can be created using information from the accelerometers on the same floor or other floors, as well as from the fitted plane using the 6DOF motion recorded on the 48th floor (see Section 2.4). In doing so, we benefit from the fact that the displacements from the slab deformations are relatively small compared to the overall deformation of the building.

#### 2.5.1. Additional Constraints for the Floors with Triaxial Sensors at Opposite Corners

**Figure 6:**
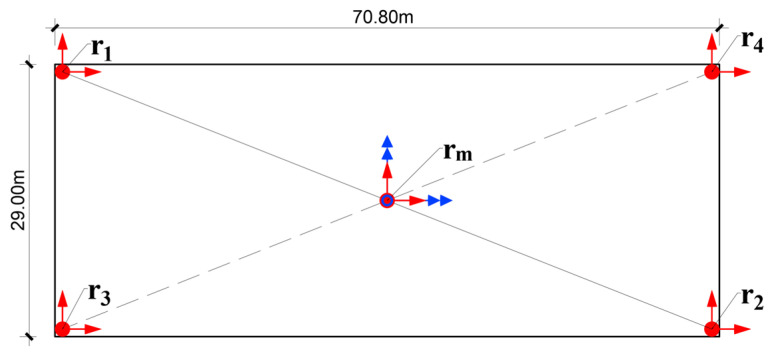
Schematic representation of a floor slab with sensors at two opposite corners.

For the floors with three sensors, two of which are at the opposite corners of one of the diagonals (see Figure 6), we construct constraints at the other corners using the measurements on the same floor, as follows. Let $r_{1}=\left(x_{1},y_{1}\right)$ and $r_{2}=\left(x_{2},y_{2}\right)$ be the positions of the existing sensors and $u\left(r_{1},t\right)$ and $u\left(r_{2},t\right)$ be their displacements at time t. Then, the midpoint position and its displacement are as follows:

(14) $$r_{m}=\frac{r_{1}+r_{2}}{2}$$

(15) $$u\left(r_{m},t\right)=\frac{u\left(r_{1},t\right)+u\left(r_{2},t\right)}{2}.$$

Assuming small motions, the rotational angles at $r_{m}$, $\psi_{x}\left(t\right)$, $\psi_{y}\left(t\right)$, and $\psi_{z}\left(t\right)$ can be approximated using finite differences as [41] shown below:

(16) $$\begin{array}{ccc} \psi_{x}\left(t\right)=\frac{1}{2}\left(\frac{\Delta u_{z}}{\Delta y}-\frac{\Delta u_{y}}{\Delta z}\right) & \psi_{y}\left(t\right)=\frac{1}{2}\left(\frac{\Delta u_{x}}{\Delta z}-\frac{\Delta u_{z}}{\Delta x}\right) & \psi_{z}\left(t\right)=\frac{1}{2}\left(\frac{\Delta u_{y}}{\Delta x}-\frac{\Delta u_{x}}{\Delta y}\right) \end{array}$$

which enables the construction of the rotation matrices $R_{x}$, $R_{y}$, and $R_{z}$, defined in Equations (10) to (12), and the combined rotation matrix

(17) $$R\left(t\right)=R_{x}\left(\psi_{x}\left(t\right)\right)R_{y}\left(\psi_{y}\left(t\right)\right)R_{z}\left(\psi_{z}\left(t\right)\right)$$

Then, the motions at the non-instrumented corners, at $r_{k}=\left(x_{k},y_{k}\right)$, k=3,4, can be estimated as

(18) $$u\left(r_{k},t\right)=R\left(t\right)\left(r_{k}-r_{m}\right)+u\left(r_{m},t\right)$$

providing additional constraints $u(r_{j},t)$ in Equation (1).

#### 2.5.2. Additional Constraints for the Floors Without Triaxial Sensors at Opposite Corners

For floors with two sensors, none of which are at the corners, we construct additional constraints using measurements recorded at the other floors along the same vertical line, using 1D spline interpolation, as follows. Let w(z) represent any one of the displacement components $u_{x}$, $u_{y}$, or $u_{z}$ as a function of the elevation z, with known values $w\left(z_{j}\right)=w_{j}$, j=1,…,N. The biharmonic equation in one dimension and the associated constraints can be written as [2]

(19) $$\begin{array}{cc} \frac{d^{4}w\left(z\right)}{dz^{4}} & =\sum_{j=1}^{N}6\alpha_{j}\delta (z-z_{j})\\ w\left(z_{j}\right) & =w_{j},\;j=1,\ldots ,N \end{array}$$

and has a solution that is a linear combination of the 1D biharmonic operator Green’s functions $\phi \left(z-z_{j}\right)$

(20) $$w\left(z\right)=\sum_{j=1}^{N}\alpha_{j}\phi \left(z-z_{j}\right)$$

in which

(21) $$\phi \left(z-z_{j}\right)={\left|z-z_{j}\right|}^{3}.$$

Then, the displacement at another height $z_{i}$ along the vertical line, where there is no sensor, can be estimated by solving the linear system of equations shown below:

(22) $$\sum_{j=1}^{N}\alpha_{j}\phi \left(z_{i}-z_{j}\right)=w_{i},\;i=1,\ldots ,N.$$

By applying this approach to each displacement component, we estimate the displacements at the non-instrumented corners providing additional constraints for the representation of the slab motion at the floors with two sensors.

#### 2.5.3. Additional Constraints for the Floor with Only One 6DOF Sensor

At the 48th floor, where measurement of 6DOF motion is available but only at one point, we showed in Section 2.4 how the slab motion can be approximated by a plane that is translating and rotating in time. The motion of the slab can also be approximated by a 2D spline interpolation, by using the plane to construct constraints at the four corners (Figure 6), using Equation (13). Then, a biharmonic spline can be fitted through the five points, creating a more natural slab displacement for the animation.

It is noted that, when using 1D spline interpolation along vertical lines, where motion is measured at some levels, the motion at the non-instrumented floors can be reconstructed and can then be used for rapid assessment of structural health during or immediately after the end of shaking.

### 2.6. Data Processing and Algorithm

The recorded data are processed as follows. The accelerations and angular velocities are extracted from the continuous streams of data, recorded at a 200 Hz sampling rate, to include, in the beginning, several seconds of ambient response prior to the arrival of the P-waves, and, at the end, the “free” response of the structure, which continues vibrating after all waves from the source have passed by the building site. After subtracting the baseline (a least squares straight line fit through the data), a Tukey window [42] in time is applied (a rectangular window tapered by cosine bell ramps at both ends; the length of each ramp is chosen to be 5% of that of the entire window). The raw angular velocities recorded by the R2 seismometers are instrument corrected with manufacturer-provided, sensor specific transfer-functions; the accelerations recorded by the EQR120 accelerometers do not require instrument correction. Then, standard data processing methods are applied, such as integration and windowing in the frequency domain [43,44].

The accelerations and angular velocities are high-pass filtered to remove the low-frequency noise [43], which is present due to the physical limitation of the sensors, which have noise levels that grow as the frequency approaches zero. The passband frequency depends on the Fourier spectrum amplitudes of the recorded motions at low frequencies and differs depending on the earthquake magnitude and distance. For most of the records in the TPYC building, a passband frequency of 0.2 Hz was sufficiently high. The records are also low-pass filtered for compatibility with the animation objective and the spatial density of sensors. We low-pass filter at 10 Hz because we aim to visualize the motion of the building, as a whole, and the band f<10 Hz contains the modes of such vibrations. This band excludes the modes corresponding to local vibrations, the visualization of which would require a much denser array of sensors. Zero phase filters should be used to avoid phase distortions, such as, e.g., Ormsby or zero phase Butterworth [42,43]. For the examples shown in this paper, we use the latter one.

The displacements and angles of rotation are computed from the recorded accelerations and angular velocities by double/single numerical integration, using linear interpolation between points, i.e., the trapezoidal rule, with zero initial conditions [44]. Following each integration, the data are again bandpass filtered to remove the low-frequency noise introduced by integration. Finally, the data are subsampled by a factor of 6, which reduces the computation time to generate the animation and results in 33 frames per second.

**Figure 7:**
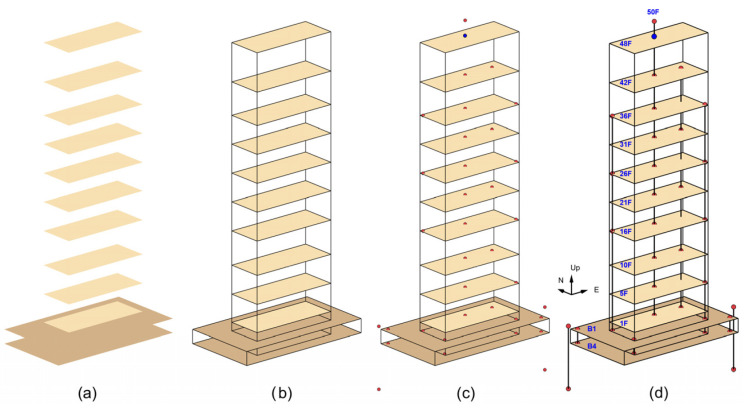
Illustration of the steps in which different elements are added to the animation: (**a**) slabs, (**b**) structural boundaries, (**c**) sensor locations, and (**d**) lines connecting neighboring sensors.

**Figure 8:**
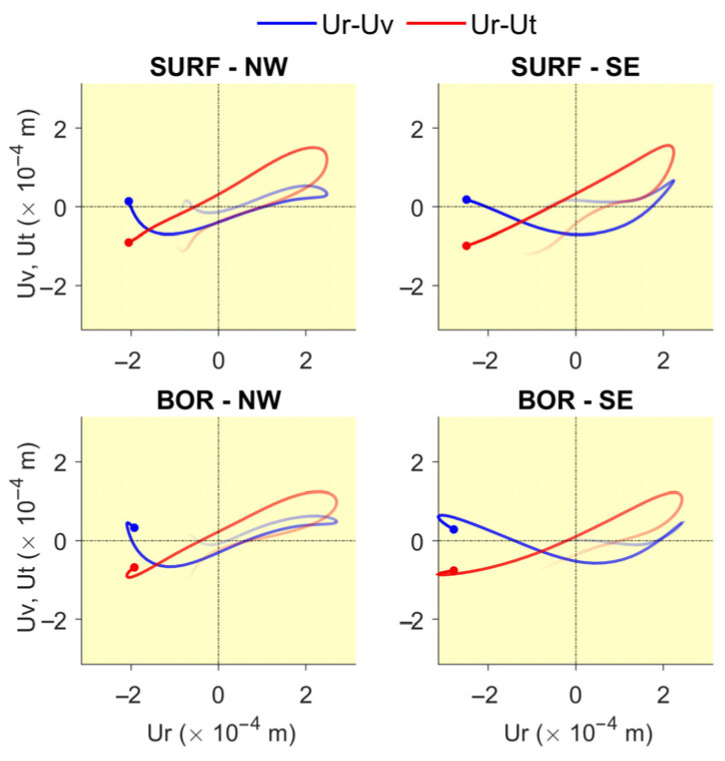
Hodograms (2 s long) showing orbits of the particle motion of the soil recorded by the sensors outside the building (SURF-NW, SURF-SE, BOR-NW, and BOR-SE) during the 2021 Shuangbai earthquake ($M_{s}$5.1, R=116 km); $U_{r}=$ radial, $U_{t}=$ transverse and $U_{v}=$ vertical components. The blue and red lines show the particle motion in vertical and horizontal planes. The dots show the motions at time t= 38.14 s.

The animation is created according to the following steps, each one adding a particular element to the animation. The steps are illustrated in Figure 7 and Figure 8.

Step 1: Add the slabs at levels 48F, 42F, 36F, 31F, 21F, 26F, 16F, 10F, 5F, 1F, −1B, and −4B, which have been sufficiently instrumented and constrained to make reconstruction of the slab motion possible (Figure 7a). The slab motion is defined at a 1 × 1 m^2^ grid of points.

Step 2: Add the physical boundaries of the structure, represented by horizontal and vertical lines, to facilitate the visualization of the structure boundaries and the sensor placements relative to the physical layout (Figure 7b).

Step 3: Add the locations of the sensors (Figure 7c). The locations of the triaxial accelerometers and 6DOF sensors are marked, respectively, by red and blue dots.

Step 4: Add lines connecting sensors on neighboring floors and add labels indicating the level (Figure 7d).

Step 5: Add hodograms showing the trajectories of particle motions recorded by the sensors of the borehole arrays at the surface (SURF-NW and SURF-SE) and at depth (BOR-NW and BOR-SE). Figure 8 shows an example of 2 s hodograms; the blue and red lines show the particle motions in vertical and horizontal planes. The horizontal axes show the radial ($U_{r}$) components and the vertical axes show the transverse ($U_{t}$) and vertical ($U_{v}$) components of motion. The dots show the motions at the time instant specified, while the thick fading lines show the trajectories during the previous 2 s. These hodograms help visualize the particle motion of the soil during the passage of Love and Rayleigh waves.

Step 6: Add a plot of time histories of the displacements at the center of the 4th basement (−4BC5), with a slider showing the current time in the animation. These displacements represent the effective input motion exciting the building.

Step 7: Choose a magnification factor for the displacements. Magnification of small amplitude response is necessary to enable viewing its features. The ratio between the peak displacement at the top and the half-width of the basement can be used as a guideline.

Step 8: Select views of the building to include in the animation. Examples are shown in the results section. The arrows under the basement show the direction of propagation of the seismic waves from the source.

## 3. Results and Analysis

**Figure 9:**
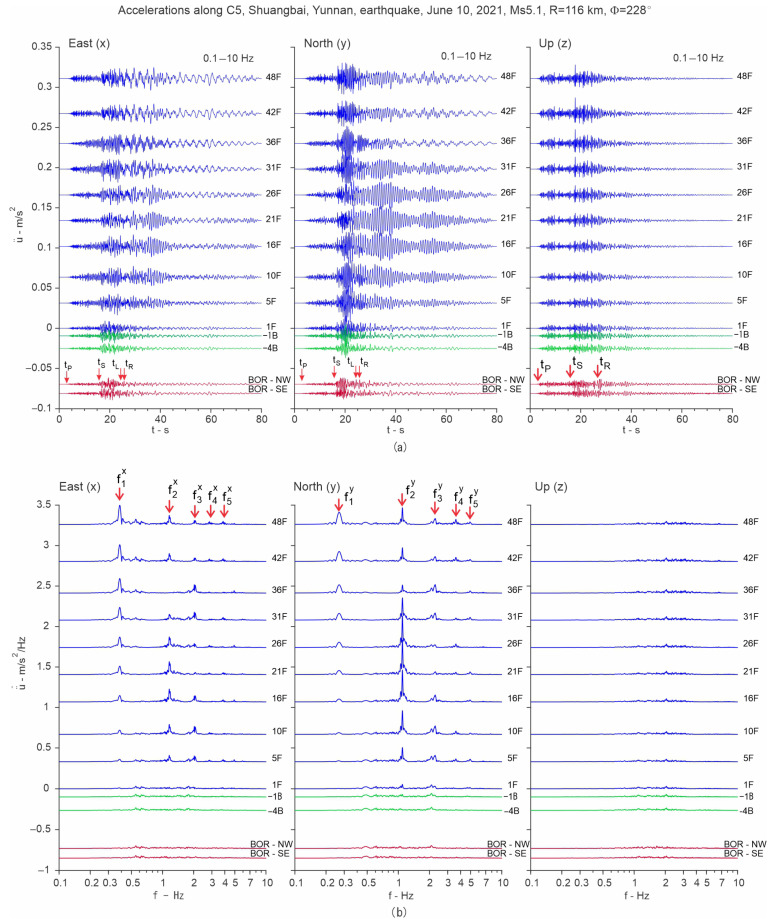
Accelerations (part **a**)) and their Fourier spectra (part **b**)) recorded during the Shuangbai earthquake of 10 June 2021 ($M_{s}$5.1, R=116 km), at different floors along column line C5 (floor centers). The motions recorded at the two borehole sensors are shown at the bottom. The motions were extracted from continuously recorded data.

**Figure 10:**
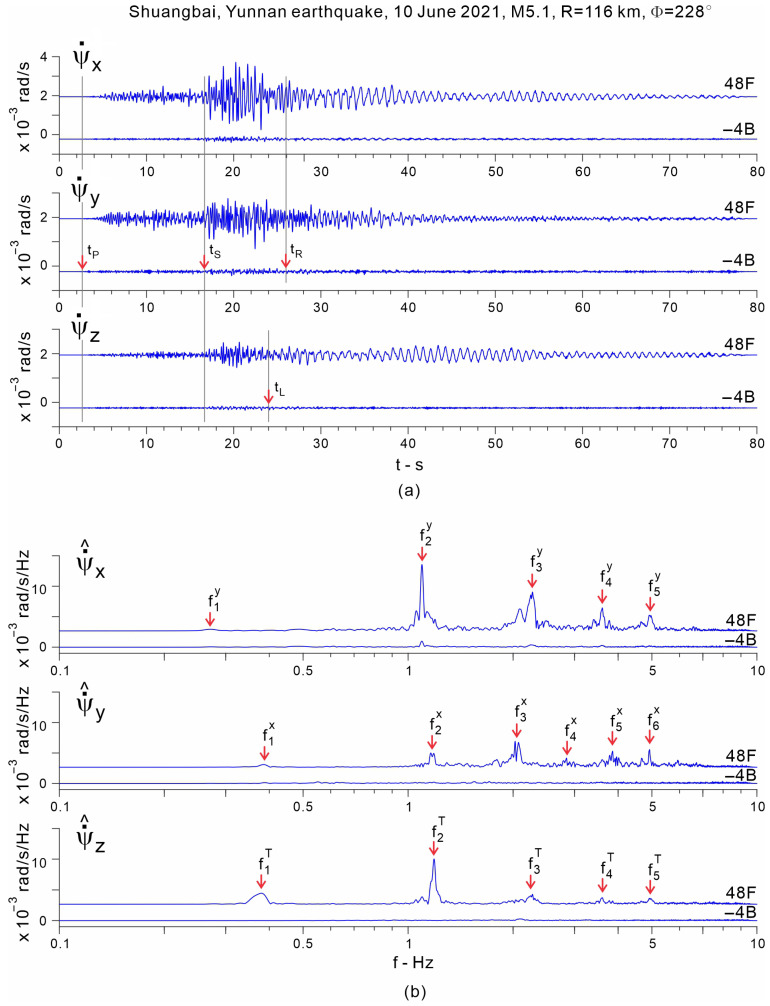
Angular velocities (part **a**)) and their Fourier spectra (part **b**)) recorded during the Shuangbai earthquake of 10 June 2021 ($M_{s}$5.1, R=116 km), at floors 48F and −4B.

The methodology is illustrated using the records of the $M_{s}$5.1 Shuangbai, Yunnan earthquake, which occurred on 10 June 2021, at 19:46 local time, at an epicentral distance R=116 km south-west of the building (back-azimuth Φ= 228°) [45]. Figure 9 shows plots of the time histories of accelerations recorded along column line C5, which is near the center of the floors, bandpass filtered between 0.1 and 10 Hz (part a)), and their Fourier spectra (part b)). The motion of the 50th floor is not shown because the accelerometer on that floor was added after this earthquake. It can be seen from the plots of the Fourier spectra that this earthquake excited at least five of the longitudinal and five of the transverse modes of vibration. Figure 10 shows such plots for the angular velocities recorded at 48F and −4B. It can be seen from plots of the Fourier spectra that most of the energy of the earthquake response is contained in the band f<10 Hz. Features of interest, such as the times of arrival of P-, S-, Love and Rayleigh waves ($t_{P}$, $t_{S}$, $t_{L}$ and $t_{R}$), are marked in the time history plots, while, in the Fourier spectra plots, the modal frequencies corresponding to the first five longitudinal (east (x)), transverse (north (y)) and torsional modes of vibration are marked ($f_{1}^{x}$ to $f_{5}^{x}$, $f_{1}^{y}$ to $f_{5}^{y}$, and $f_{1}^{T}$ to $f_{5}^{T}$).

**Figure 11:**
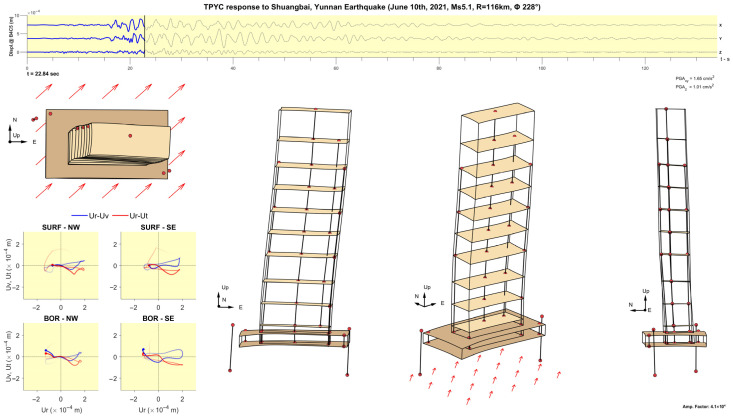
Snapshot at t= 22.84 s from an animation of the recorded response in the TPYC during the Shuangbai earthquake of 10 June 2021 ($M_{s}$5.1, R=116 km), amplified by a factor of 4.1 × 10^4^.

**Figure 12:**
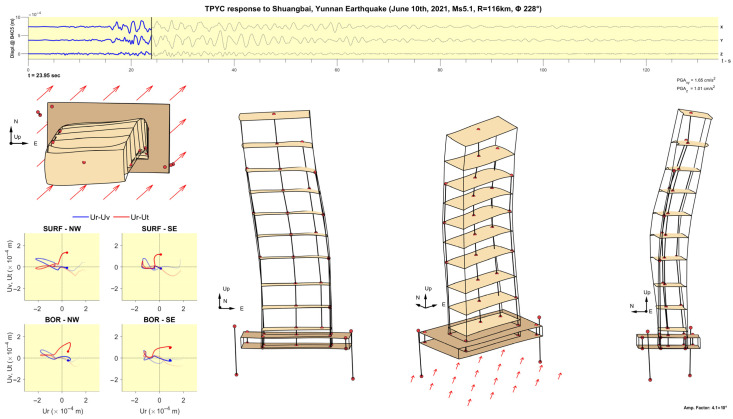
Same as Figure 11 but at time t= 23.95 s.

**Figure 13:**
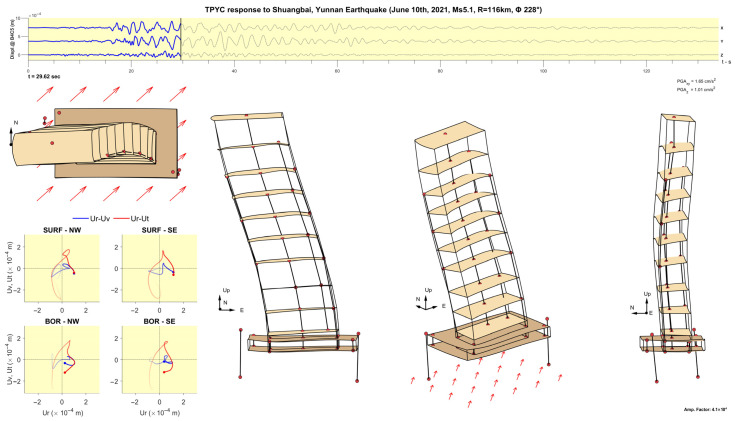
Same as Figure 11 but at time t= 29.62 s.

**Figure 14:**
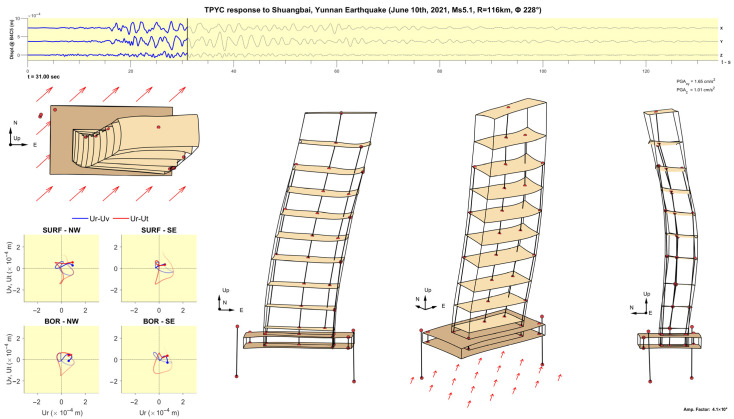
Same as Figure 11 but at time t= 31.00 s.

**Figure 15:**
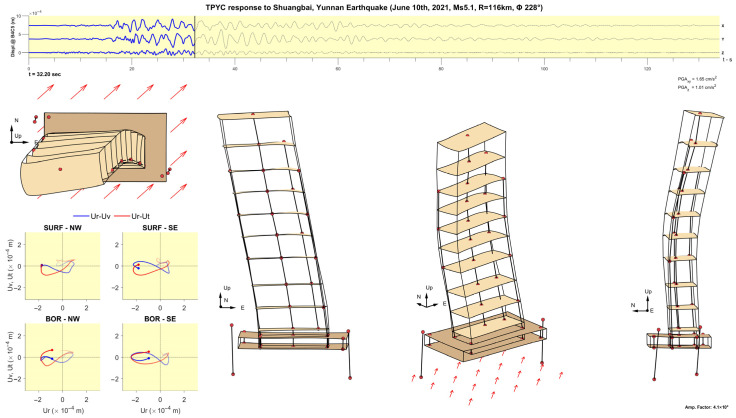
Same as Figure 11 but at time t= 32.20 s.

**Figure 16:**
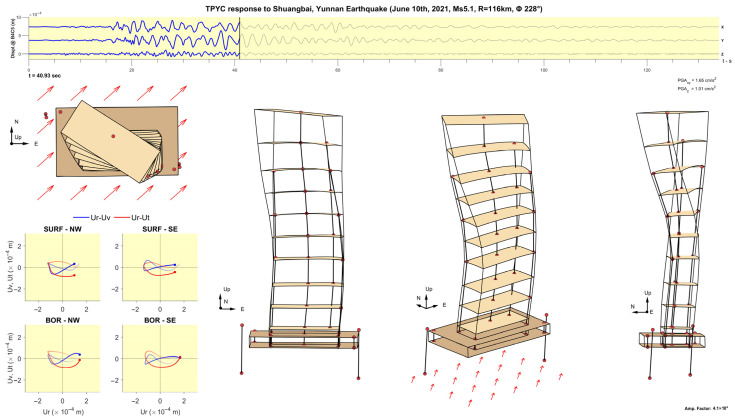
Same as Figure 11 but at time t= 40.93 s.

**Figure 17:**
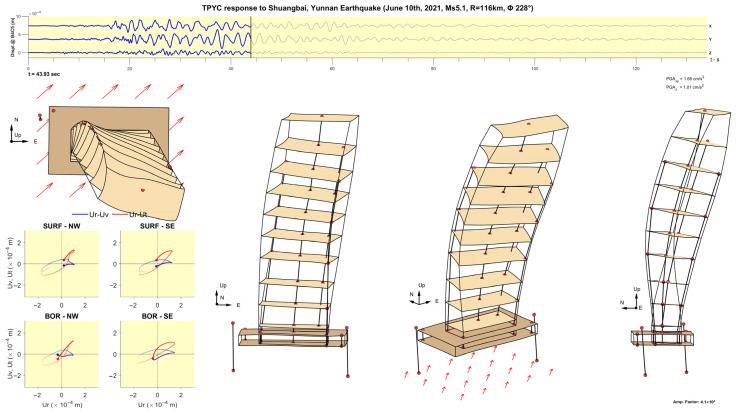
Same as Figure 11 but at time t= 43.93 s.

**Figure 18:**
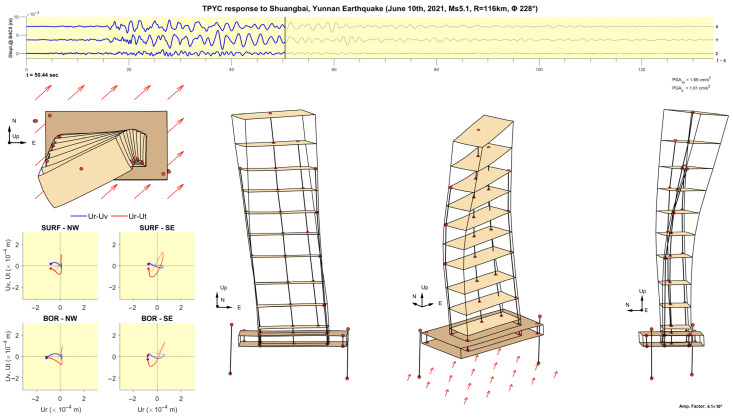
Same as Figure 11 but at time t= 50.44 s.

Figure 11, Figure 12, Figure 13, Figure 14, Figure 15, Figure 16, Figure 17 and Figure 18 show screenshots of the animation, which can be played on YouTube [46], at eight instants, with the displacements magnified by a factor of 4 × 10^4^. The animation shows four views of the building: top, south, south-west, and west, the time history of the displacements in the 4th basement (at C5), and hodograms of the particle motion of the soil recorded by the surface and downhole sensors. It is evident from the deformations of the structure that the earthquake excited the higher modes of vibration and that the torsional vibrations of the tower were significant. The basement deformation during the passage of the earthquake waves is also evident, as well as the motion of the tower relative to the basement, a phenomenon that we termed “tower–basement” interaction. This confirms the findings of our previous studies on the effects of soil–structure interaction (SSI) on the building’s response based on a detailed finite element model [39]. The hodograms show that the particle orbits of the surface waves are more complex than the theoretical ones, such as the retrograde elliptical motion during the passage of Rayleigh waves in a uniform half-space.

Animations of the response to five earthquakes, including Shuangbai, can be viewed on YouTube at the @TPYC-seismic channel [46]. The other four earthquakes are Yangbi, Yunnan, on 21 May 2021 ($M_{s}$6.4, R=295 km); Burma on 29 July 2021 ($M_{s}$5.7, R=730 km); Kanding (Luding), Sichuan, on 26 January 2023 ($M_{s}$5.6, R=512 km); and Xishan, Yunnan, on 27 January 2023 ($M_{s}$2.1, R=10 km).

## 4. Discussion and Conclusions

In the presented results, the motion of the floor slabs has been animated only for the instrumented floors, i.e., for 10 out of 50 floors above ground, and 2 out of 4 basement floors, in order to avoid clutter and enable a clear view of the slab motions and used for post-earthquake visualization of the response. The method, however, can be extended to the other floors, by creating “recordings” on these floors by bi-harmonic spline interpolation of the motions along the vertically aligned sensors, which can then be used as constraints for the 2D bi-harmonic spline interpolation of the slab motion. In general, the quality of the animations produced by the proposed method will increase with increasing spatial density of the sensors.

In this paper, the animations were created after the complete records had been collected. However, the method is also suitable for real time application, benefiting from the computational efficiency of the interpolation. The interpolated motions can then be used for *real time* monitoring and rapid assessment of the structural health, based, e.g., on published relationships between interstory drifts and damage levels [47].

The computation of the dynamic response of tall structures using numerical models has evolved from the vibrational approach, which, in the linear range of response uses mode superposition, to the alternative wave propagation approach [48,49,50,51,52], which has recently gained considerable interest but is still rarely used in practical engineering design work. To this end, the animations created by the proposed method provide convincing evidence about the wave nature of the seismic response of tall buildings.

It is concluded that the proposed method is a powerful tool for visualization of recorded 3D earthquake response of a structure by a dense sensor array, which provides invaluable insight into how actual buildings respond to earthquakes. Visualization provides opportunities for the validation of numerical model-based simulations, e.g., related to wave propagation vertically through the structure and soil–structure interaction, and application to real time structural health monitoring.

## Figures and Tables

**Table 1:** Coordinates of the sensor locations in the TPYC.

Sensors Above Ground Level					Sensors Below Ground Level				
Level	Location	X (m)	Y (m)	Z (m)	Level	Location	X (m)	Y (m)	Z (m)
50F	C5	0	0	231.8	−1B	PBP16	51.4	−25.75	−6.7
48F	C5	0	0	219.7		PNP2	−51.1	27.3	−6.7
42F	C8	25.8	0	188.9		D9	35.1	8.3	−6.7
	C5	0	0	188.9		A1	−33.5	−16.5	−6.7
36F	A9	35.1	−16.5	162.6		A9	35.1	−16.5	−6.7
	D1	−33.5	8.3	162.6		D1	−33.5	8.3	−6.7
	C5	0	0	162.6		C5	0	0	−6.7
31F	C8	25.8	0	140.1	−4B	PBP16	51.4	−25.75	−17.8
	C5	0	0	140.1		PNP2	−51.1	27.3	−17.8
26F	A9	35.1	−16.5	117.3		D9	35.1	8.3	−17.8
	D1	−33.5	8.3	117.3		A1	−33.5	−16.5	−17.8
	C5	0	0	117.3		A9	35.1	−16.5	−17.8
21F	C8	25.8	0	94.8		D1	−33.5	8.3	−17.8
	C5	0	0	94.8		C5	0	0	−17.8
16F	A9	35.1	−16.5	72	SURF	NW	−63.8	22.85	0
	D1	−33.5	8.3	72		SE	56.6	−23.85	0
	C5	0	0	72	BOR	NW	−63.8	22.85	−49.2
10F	C8	25.8	0	45		SE	56.6	−23.85	−49.2
	C5	0	0	45					
5F	A9	35.1	−16.5	22.2					
	C5	0	0	22.2					
1F	C8	25.8	0	0					
	C5	0	0	0					

## Data Availability

The raw data used for the animations presented in this article are not readily available because they are part of an ongoing study. Requests to access the datasets should be directed to the second author, M.T. The video files of the animations are available on the @TPYC-seismic YouTube channel.
